# Advanced tubal pregnancy at 34 weeks with eclampsia and HELLP syndrome: a case report and literature re

**DOI:** 10.1186/s12884-023-05469-w

**Published:** 2023-03-04

**Authors:** Yang Liu, Xiaoping Xu, Qian Liu, Xiaolan Luo, Bin Cai, Jingyuan He, Ruiqian Liu

**Affiliations:** 1 Department of Obstetrics and Gynecology, Deyang People’s Hospital, Deyang, 61800 Sichuan China; 2 Department of Pathology, Deyang People’s Hospital, Deyang, 61800 Sichuan China; 3 Department of Radiology, Deyang People’s Hospital, Deyang, 61800 Sichuan China

**Keywords:** Tubal pregnancy, Pre-eclampsia, Eclampsia, HELLP syndrome

## Abstract

**Background:**

Tubal ectopic pregnancies in the late stages of pregnancy are uncommon, and reports on their complications are scarce. We present the case of a woman who had a tubal ectopic pregnancy at around 34 weeks and developed severe pre-eclampsia complications.

**Case:**

A 27-year-old woman presented to our hospital several times with vomiting and convulsions. A physical exam revealed hypertension, scattered ecchymosis, and a large abdominal mass. A computed tomography (CT) scan performed in an emergency revealed an empty uterus, a stillbirth baby in the abdominal cavity, and a crescent-shaped placenta. Blood tests revealed that the patient had a low platelet count and clotting dysfunction. Laparotomy confirmed advanced right fallopian tube pregnancy without rupture, and salpingectomy was performed. Pathological examination revealed a significantly thickened tubal wall, adhesion of the placenta, and poor placental perfusion.

**Conclusion:**

The unusually thickened muscular layer of the tube may be one of the reasons for tubal pregnancy progressing to an advanced stage. Placenta adhesion and the special site to which it is attached reduce the risk of rupture. The detection of a crescent-shaped placenta on imaging may aid in the accurate diagnosis, distinguishing between abdominal and tubal pregnancy. Women with advanced ectopic pregnancy are more likely to develop pre-eclampsia and have poorer maternal-fetal outcomes. These negative outcomes may be influenced by abnormal artery remodeling, villous dysplasia, and placental infarction.

## Background

Tubal pregnancy, which accounts for 94% of all ectopic pregnancies [1], is always treated early for the typical clinical manifestations of amenorrhea, bleeding, and abdominal pain, as well as massive intraperitoneal hemorrhage. Almost 80% of them have ruptured fallopian tubes during early pregnancy due to a thin wall [2, 3]. Therefore, there are few reports of late-stage cases, particularly with complications that always occur in normal pregnancy. We present the case of a woman with tubal pregnancy at 34 weeks who was admitted to our hospital for pre-eclampsia with a series of severe complications rather than abdominal pain or massive hemorrhage.

## Case report

On August 18, 2022, a 27-year-old woman, gravida 3, para 1, was referred to our hospital with complaints of vomiting and convulsions. She was unconscious and under the influence of intravenous diazepam when she arrived. Her last menstrual period was unknown; however, her relatives reported an irregular menstrual pattern for 8 months. Her first child died at 27 weeks for unknown reasons, and she gave birth to a live male baby in our hospital a year later. Albuminuria was confirmed in both pregnancies without hypertension. Her family history was unremarkable.

Her blood pressure, respiratory rate, pulse rate, and temperature were all 190/139 mmHg, 25/min, 150/min, and 36.8 °C, respectively, on physical examination. Her skin was covered in ecchymosis, and she had significant edema in the lower part of her shank. A soft mass with a fetus of more than 8 months and no heart tones was discovered during an abdominal examination using Doppler ultrasound. Without dilation, a vaginal examination revealed a long, thick cervix.

Her laboratory parameters were abnormal; alanine aminotransferase, aspartate aminotransferase, lactate dehydrogenase, amylase, and creatinine levels were elevated up to 195 U/L(normal reference value:7–40 U/L), 755 U/L(13–35 U/L), 2355 U/L(120–250 U/L), 227 U/L(0–100 U/L), and 341.6 μmol/L(41–73 μmol/L), respectively. Blood clotting test revealed coagulation dysfunction, with an activated partial thromboplastin time of 24.00 s, fibrin degradation product level of 94.10 mg/L, and D-dimer level of 22.74 mg/L. Her hemoglobin levels were normal, but her platelet count had dropped to 46 × 10^9^/L, and her proteinuria was + 2.

**Fig. 1:**
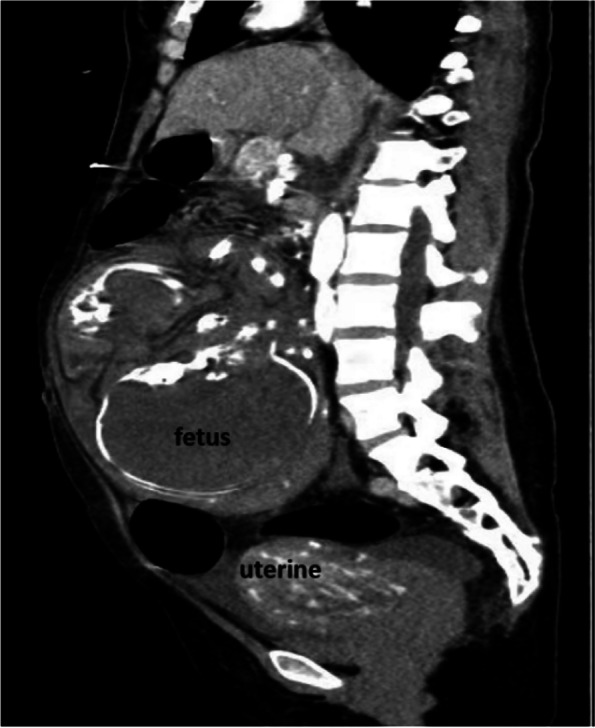
Abdominal CT revealing an empty uterus and a longitudinal fetus outside

**Fig. 2:**
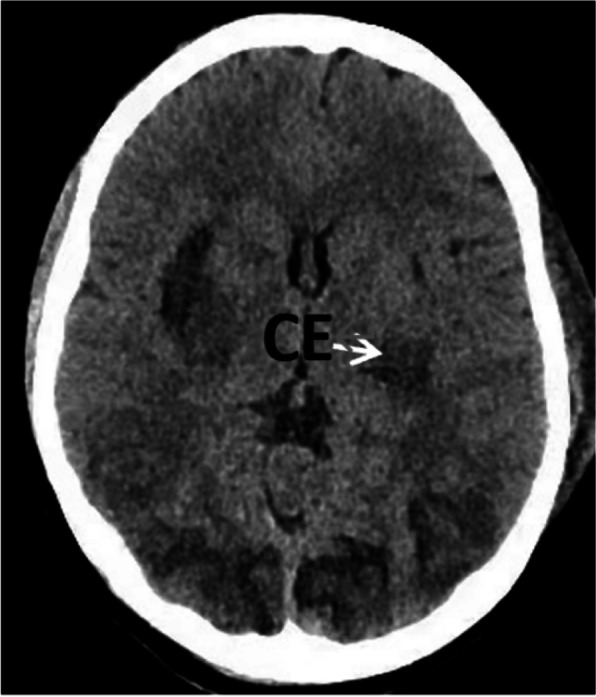
Head CT revealing extensive narrowing of the cerebral sulcus and a decrease in the density of the bilateral basal ganglia and centrum semiovale, indicating cerebral edema

An abdominal ultrasound revealed an empty uterus and an extra-uterine fetus with no heartbeat or movement; the fetus’ estimated gestational age was 34 weeks. Following contrast-enhanced CT, these findings were confirmed (Fig. 1). An abdominal pregnancy was thus suspected. A CT scan also revealed mild cerebral edema (Fig. 2), hydrocholecystis, and an enlarged pancreas.

**Fig. 3:**
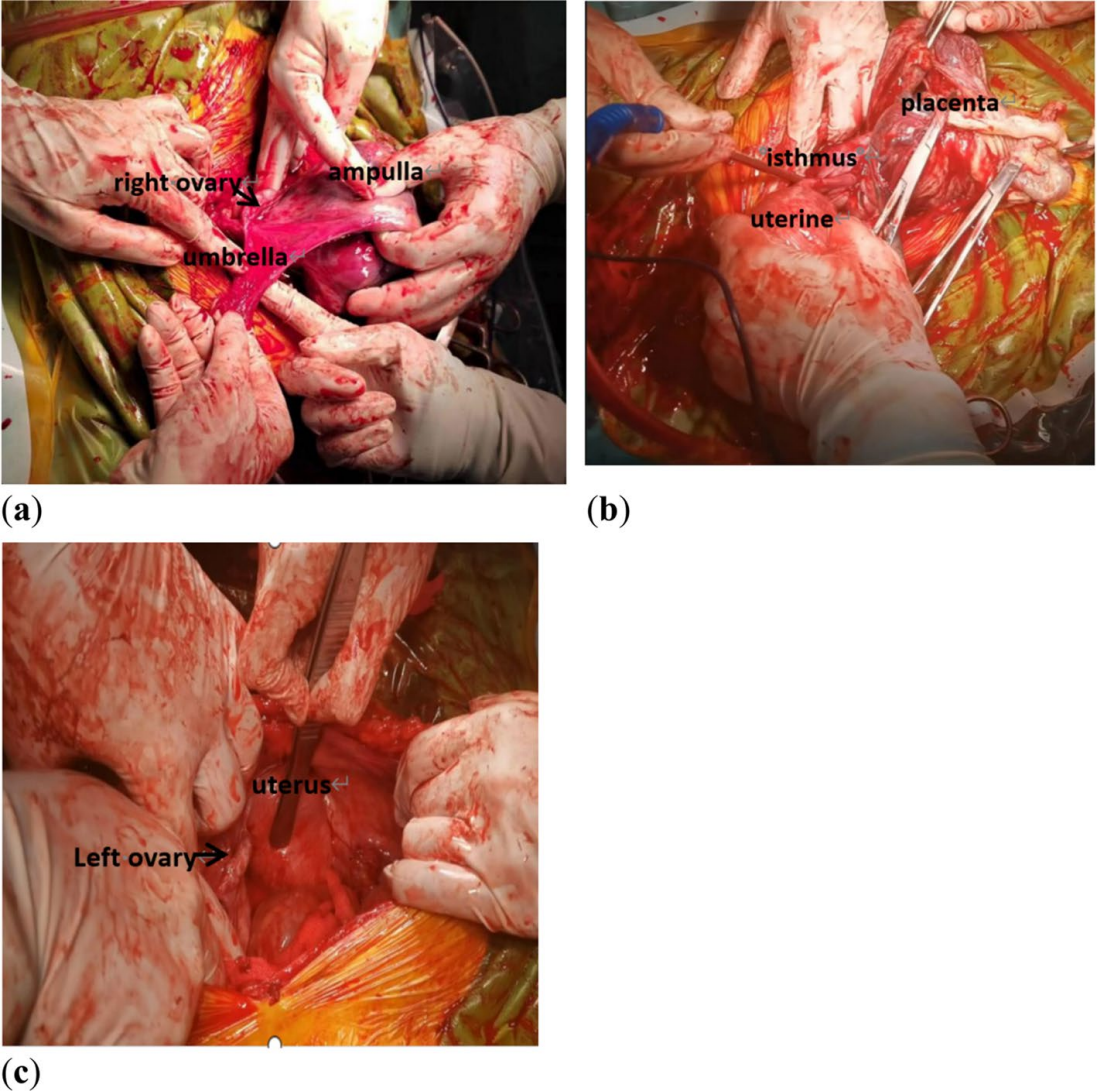
The placenta was located in the enlarged ampulla of the right fallopian tube, which was connected to the ipsilateral horn via the thickened and prolonged isthmus. The left fallopian tube, bilateral ovaries, and uterus were normal in appearance

An emergency laparotomy was performed based on the findings of abdominal pregnancy and eclampsia. The abdomen was entered without incident while under general anesthesia. A female stillbirth weighing 2.1 kg was delivered from the intact amniotic sac, which was completely enveloped in the right unruptured salpinx. The uterus, left fallopian tube, and ovaries appeared normal (Fig. 3). The placenta measured 15 × 10 × 2 cm in size and covered the majority of the ampulla cavity. Due to the dense adhesion, the surgeons were unable to separate it from the tissue. A right salpingectomy was carried out. The total amount of blood lost was 800 mL. Six units of cryoprecipitate and four units of packed fresh frozen plasma were transfused during the procedure. Following the operation, she was transferred to the intensive care unit. To control blood pressure, nitroglycerin and urapidil were administered intravenously via a micropump. Additionally, concentrated platelets were infused to boost counts, and ceftriaxone was given to prevent infection. Fortunately, the patient regained consciousness 2 days later and was released 18 days later. A one-month follow-up visit revealed normal blood pressure and no albuminuria.

**Fig. 4:**
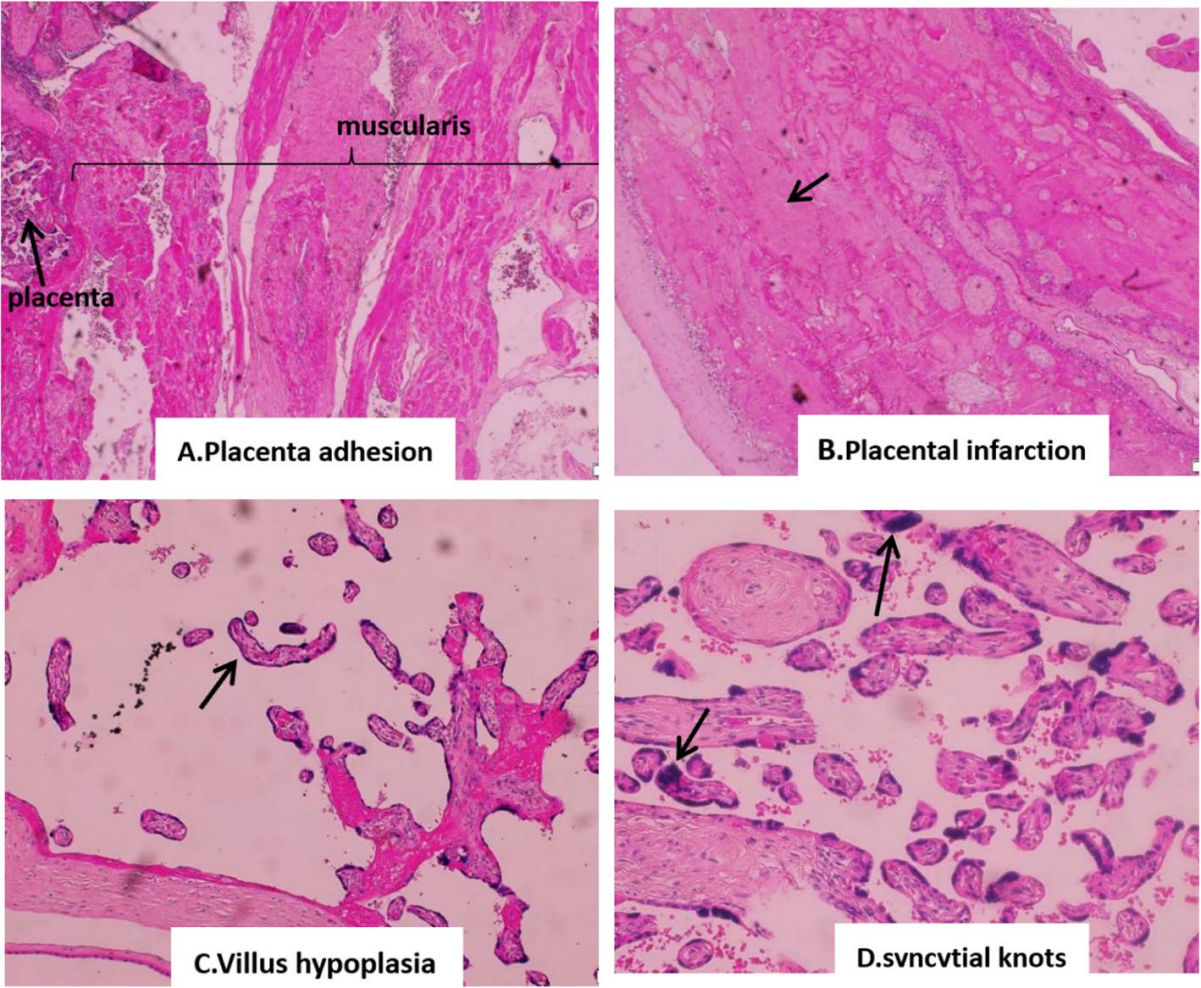
Pathological findings under a microscope revealing several remarkable manifestations and no artery remodeling. **A** The muscular layer of the oviduct wall was obviously thickened, the decidual plate placenta was absent, and the placenta was adhering directly to the musculature; **B** Most trophocytes had lost their nuclei, with inadequate blood supply; **C** Several terminal villi had become elongated, less branched, and sparse and some did not have vessels; **D** Aggregation of syncytiotrophoblast nuclei was along villi to form syncytial knots

The myometrium of the tube was significantly thickened, and the myocytes were hypertrophic, according to histopathogical examination. Furthermore, the placenta had adhered directly to the musculature due to the absence of the decidual plate. There were no trophoblasts invading the inner wall of the poorly remodelled artery, and a large number of villus cells had lost their nuclei, with syncytiotrophoblast nuclei aggregating along the villi, known as syncytial knots; some terminal villi were elongated, less branched, and sparse, and some did not have vessels (Fig. 4).

## Discussion and conclusion

### Why some tube pregnancies can reach the second or third trimester?

The tube wall struggles to accommodate the gradually increasing sac and trophoblast erosion, frequently resulting in rupture at 7.2 ± 2.2 weeks [4]. However, a few cases of advanced gestation have been reported: 14 [5], 15 [6], 17 [7], 29 [8], 42 [9], 45, and 50 [10] weeks. In our case, an 8-month-old fetus and appendage were discovered in the intact tube cavity and were evaluated by CT and ultrasound at 34 weeks. According to Lichtenstein and Schumann, placental development occurs at a point along the mesosalpinx attachment line, and adequate blood supply and greater elasticity of the tube wall are important factors. Christopher proposed that adhesion could have also been a source of blood supply for the sac [11]. In our case, the patient did not have synechia surrounding the oviduct; however, her placenta almost completely covered the inner side of the tube, including the location mentioned by Lichtenstein and Schumann. We also noticed that the thickness of the tube wall was approximately 0.5 cm, which was significantly thicker than normal; we suspected that these two factors were the primary causes of the no rupture. Pathological examination revealed only a few hypertrophic myocytes, which resembled the myometrium in a normal pregnancy.

### How to distinguish tubal pregnancy from abdominal pregnancy in the advanced trimester?

**Fig. 5:**
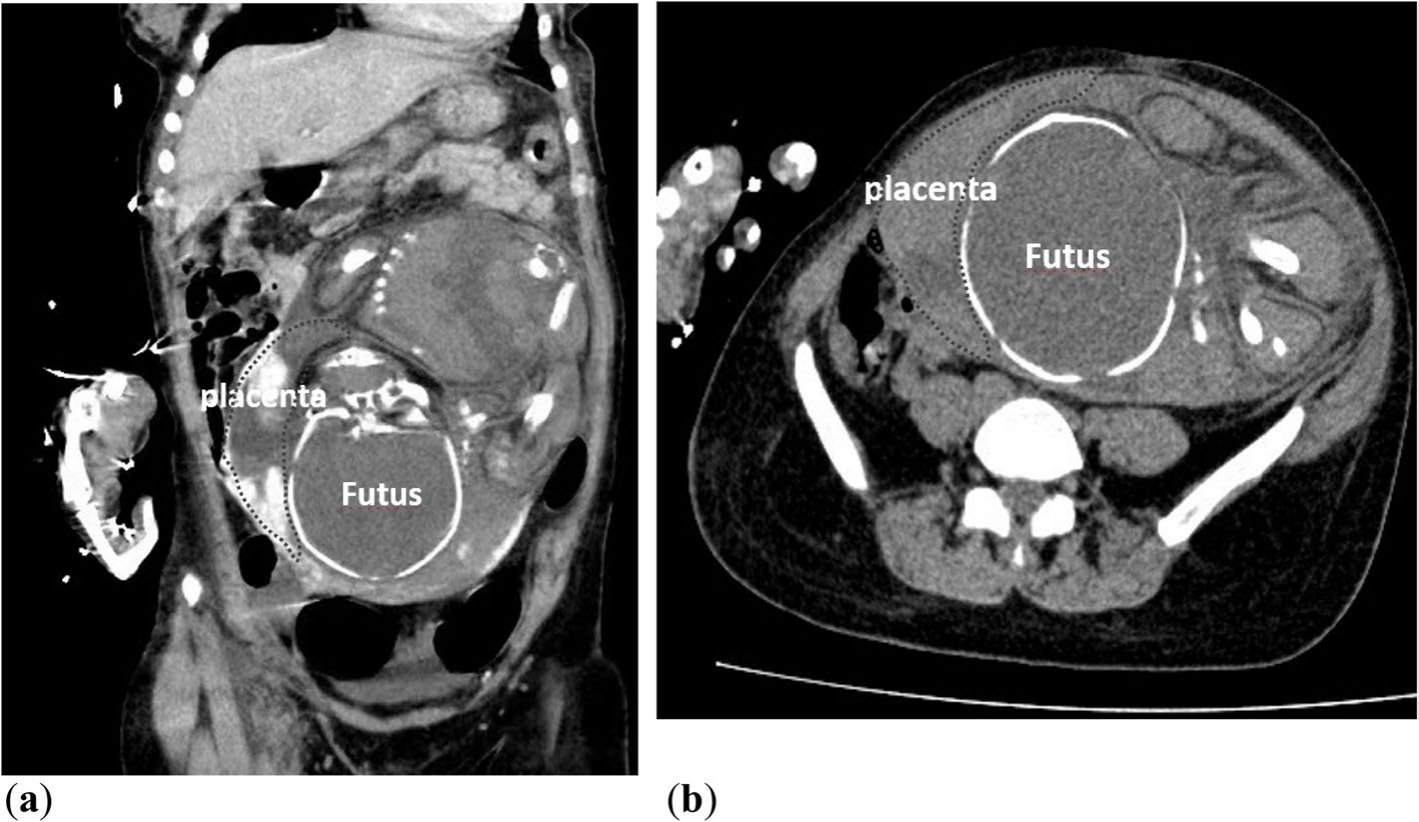
Sagittal- or coronal-view CT revealing crescent placenta

A paraovarian mass, thickened fallopian tube, uterine endometrium with a ≤ 10 mm thickness, and a high echo are all indicators of an early tubal pregnancy [12]. Based on these considerations, early diagnosis becomes simple. On the other hand, sonographic findings based on diagnosis are less useful in the advanced stage and always mimic abdominal pregnancy until laparotomy is performed for similar clinic manifestations and imaging characteristics [13, 14]. In our case, the woman did not have abdominal pain and mistook her irregular bleeding for a menstrual disorder, causing her to miss her early ultrasonic examination. When she was admitted to the hospital in late pregnancy, her condition was misdiagnosed as abdominal pregnancy until the surgeon entered his abdominal cavity. According to Joseph et al., a well-defined gestational sac with a crescent placenta most likely represented a tubal pregnancy in the second trimester. In contrast, a misshapen sac with a flattened placenta most likely represented an abdominal pregnancy [14]. We also discovered the same crescent-shaped placenta in the MRI figure reported by Baruah [13]. After that, we reviewed our CT images and discovered the same crescent-shaped placenta (Fig. 5). While the placenta is obviously attached to the inner side of the fallopian tube, it is slightly curled inward to accommodate the round tube cavity, giving the placenta the appearance of a curved moon when viewed longitudinally. In an abdominal pregnancy, however, the placenta does not have to be curved because it usually lies flat on the surface of the omentums, bowel, or mesentery. By using different CT or MRI imaging, we can distinguish tubal pregnancy from an abdominal pregnancy. These findings support our recommendation of CT as an alternative technique for distinguishing between these two advanced extra-uterine pregnancies without considering the fetus. In emergency rooms, CT scans are more easily obtained than MRIs.

### What is the status of pre-eclampsia in ectopic pregnancies?

Worldwide, 3-5% of intrauterine pregnancies have pre-eclampsia [15]. Except for a few case reports, there are no statistics on ectopic pregnancy due to its low incidence. Using the keywords “ectopic/extra-uterine/tubal/abdominal/residual horn pregnancy” and “hypertensive disorder of pregnancy/pre-eclampsia/eclampsia/HELLP syndrome/thrombocytopenia/coagulation dysfunction” in PubMed, we collected 17 English reports, including 26 cases with hypertensive disorder of pregnancy or pre-eclampsia and relative complications” (Table 1) [16–32]. In summary, almost all patients had pre-eclampsia (25, 96.15%), with 24 having severe pre-eclampsia (92.31%) and 9 having eclampsia (34.62%), both of which were significantly higher than those observed in normal pregnancies as reported by Baig et al. (34.5 and 8.5%, respectively) [33]. However, until ours, no case of HELLP syndrome has been reported. In addition, there were 15 fetal or neonatal deaths, 7 had intrauterine growth restriction, and 12 were low-birth-weight infants, except for 9 live births and 2 unknown births. Ectopic pregnancy with pre-eclampsia appears to have negative consequences for both the mother and the fetus.

**Table 1:** Cases of ectopic pregnancy with the hypertensive disorder

Author	Mother						Fetus	
	Gestation	Sac site	Eclampsia	Severe PE features	Postoperative complications	0utcomes	Weight(kg)	outcomes
1.EDWARD Ar, LEN,1928	32^+6^weeks	Abdominal cavity	yes	no	Placental retention phlogosis	recovery	1.93	died (8 h later)
2.DR.A.F.LASH,1938	33^+4^weeks	Abdominal cavity	no	SP ≥ 160 or DP ≥ 110	no	recovery	2.55	alive
3.C.B. PRIDE,1942	18 weeks	ovary	yes	no	hematorrhea	died	unknown	stillbirth
	term	tube	yes	no	no	died	3	stillbirth
	unknown	unknown	yes	no	no	died	unknown	stillbirth
	6 + m	Abdominal cavity	yes	no	no	recovery	unknown	stillbirth
	8 m	Abdominal cavity	yes	no	no	recovery	unknown	stillbirth
	term	Abdominal cavity	yes	no	psychosis	recovery	1.93	died (8 h later)
	8 m	Abdominal cavity	yes	no	hematorrhea	died	unknown	unknown
4.Fred Benjamin,1961	34^+3^weeks	Abdominal cavity	no	SP ≥ 160 or DP ≥ 110	no	recovery	2.27	alive
	36 weeks	Abdominal cavity	no	SP ≥ 160 or DP ≥ 110	no	recovery	2.41	alive
5.M Felbo,1966	37 weeks	abdominal cavity	no	SP ≥ 160 or DP ≥ 110	hematorrhea	recovery	3	alive
6.JOHN F. J.CLARK,1967	38–39 weeks	abdominal cavity	no	SP ≥ 160 or DP ≥ 110 headache	no	recovery	unknown	stillbirth
	36–38 weeks	abdominal cavity	no	SP ≥ 160 or DP ≥ 110	no	recovery	unknown	unknown
7.W.G. Paterson,1975	37^+4^weeks	broad ligament	no	SP ≥ 160 or DP ≥ 110	hematorrhea	recovery	2.03	alive
8.R.W.Baehler,1975	term	abdominal cavity	no	no	no	recovery	1.96	alive
9.K.J.ANDERTO,1976	35^+4^weeks	abdominal cavity	no	SP ≥ 160 or DP ≥ 110	no	recovery	2.1	stillbirth
10.C.C.Ekwempu,1979	36 + weeks	abdominal cavity	no	no(2+ proteinuria)	no	recovery	3.15	Died (4 days later)
11.J.MOODLEY,1987	39 weeks	abdominal cavity	no	SP ≥ 160 or DP ≥ 110 Headache nausea	no	recovery	3.1	alive
	26 weeks	abdominal cavity	no	SP ≥ 160 or DP ≥ 110	no	recovery	0.6	Died (several hours later)
12.J.O.Emembolu,1989	26 weeks	abdominal cavity	no	SP ≥ 160 or DP ≥ 110 headache blurred vision	no	recovery	unknown	unknown
13.Walter F.Piering,1993	37 weeks	abdominal cavity	no	SP ≥ 160 or DP ≥ 110	no	recovery	2.8	stillbirth
14.Hiroyuki Seki,1997	30 weeks	ovary	no	SP ≥ 160 or DP ≥ 110 renal disfunction	no	recovery	1.40	stillbirth
15.Helga M. de Muelenaere,2003	33 weeks	abdominal cavity	no	SP ≥ 160 or DP ≥ 110 thrombocytopenia	no	recovery	1.6	stillbirth
16B.A.Ekele,2007	8 m	abdominal cavity	yes	SP ≥ 160 or DP ≥ 110	hematorrhea	recovery	1.1	stillbirth
17.Hailu,2017	37 + 2 weeks	abdominal cavity	no	headache blurred vision	no	recovery	1.8	alive

Although the pathogenesis is complex, most researchers believe that impaired extravillous trophoblast infiltration, which results in abnormal spiral artery remodeling, causes maternal vascular malperfusion and resistance elevation [34, 35]. What about in the case of a tubal pregnancy? For the first time, the pathological characteristics of the placenta in an ectopic pregnancy with pre-eclampsia were revealed in our report. Because the decidua was missing, the placenta attached directly to the musculature, as Radaelli reported [36]. The placenta was severely infarcted, and the terminal villi were underdeveloped. Because of endometrial agenesis of the salpinx tissue and insufficient blood supply, artery remodeling may have been abnormal, resulting in poor placental perfusion and possibly causing pre-eclampsia in women with advanced tubal pregnancy.

Hypoperfusion of the placenta leads to placental ischemia and placental factors release into the maternal circulation. These events trigger a series of immune responses and lead to various complex and serious manifestations [36–39]. It is not known if there will be a more intense response in women with tubal pregnancy with less placental perfusion. Except for eclampsia and HELLP syndrome, this women’s brain, gallbladder, pancreas, and kidneys were all involved. As mentioned above, women with ectopic pregnancy with the hypertensive disorder may have a higher risk of developing severe conditions. However, Hailu et al. and Han et al. reported that there is no clue to manifest this relationship [32, 40], and more clinical case studies and explorations are needed.

Advanced tubal pregnancy is uncommon. The unusually thickened muscular layer, placenta adhesion, and the unique location of placenta implantation could be the cause. The identification of crescent-shaped placenta on imaging is valuable in the accurate diagnosis, distinguishing from abdominal pregnancy. Physicians should also keep an eye out for complications; women with advanced-stage ectopic pregnancy are more likely to develop pre-eclampsia and have poorer maternal-fetal outcomes. These negative outcomes may be influenced by abnormal artery remodeling, villous dysplasia, and placental infarction.

## Data Availability

The data presented in this study are available on request from the corresponding author.

## Abbreviations

HELLP: Hemolysis, elevated liver enzymes, and low platelets
MRI: Magnetic resonance imaging
CT: Computed tomography
